# Subcytotoxic Exposure to Avobenzone and Ethylhexyl Salicylate Induces microRNA Modulation and Stress-Responsive PI3K/AKT and MAPK Signaling in Differentiated SH-SY5Y Cells

**DOI:** 10.3390/ijms27031134

**Published:** 2026-01-23

**Authors:** Agnese Graziosi, Luca Ghelli, Camilla Corrieri, Lisa Iacenda, Maria Chiara Manfredi, Sabrina Angelini, Giulia Sita, Patrizia Hrelia, Fabiana Morroni

**Affiliations:** 1 Department of Oncology and Hemato-Oncology, University of Milan, Via Festa del Perdono 7, 20122 Milan, Italy; agnese.graziosi@unimi.it; 2 Department of Pharmacy and BioTechnology—FaBiT, Alma Mater Studiorum University of Bologna, Via Irnerio 48, 40126 Bologna, Italy; luca.ghelli3@unibo.it (L.G.); s.angelini@unibo.it (S.A.); fabiana.morroni@unibo.it (F.M.); 3 Clinical Pharmacology Unit, IRCCS Azienda Ospedaliero-Universitaria di Bologna, Via Giuseppe Massarenti 9, 40138 Bologna, Italy

**Keywords:** endocrine disruptors, microRNA, UV filter, neurotoxicity, avobenzone, ethylhexyl salicylate

## Abstract

Avobenzone (AVO) and ethylhexyl salicylate (EHS) are widely used organic UV filters with distinct photochemical properties and reported biological effects. Experimental and predictive evidence suggests that some lipophilic UV filters may reach systemic circulation and potentially cross the blood–brain barrier (BBB), raising concerns about possible central nervous system effects, although direct evidence for AVO and EHS remains limited. This study evaluated the effects of subcytotoxic concentrations (0.01–1 µM) of AVO and EHS on differentiated SH-SY5Y human neuroblastoma cells, focusing on early stress-related molecular responses. Cell viability and reactive oxygen species production were not significantly affected at any tested concentration. Integrated analyses of microRNA, gene, and protein expression revealed modest and variable modulation of miR-200a-3p and miR-29b-3p. Western blot analysis showed increased phosphorylation of AKT and ERK, no significant changes in mTOR activation, and an increased Bax/Bcl-2 ratio. Overall, these findings indicate that AVO and EHS trigger an early stress-adaptive response involving PI3K/AKT and MAPK/ERK signaling and modulation of apoptosis-related pathways. Such responses reflect a dynamic balance between cellular adaptation and pro-apoptotic signaling, which may become relevant under prolonged or higher-intensity exposure conditions.

## 1. Introduction

Endocrine disruptors (EDs) are exogenous chemicals, including industrial cosmetic and pharmaceuticals substances, agricultural products, and heavy metals that interfere with the synthesis, secretion, transport, metabolism, or elimination of physiological hormones. EDs impair the hormonal activity by mimicking hormones, triggering inappropriate responses at the wrong times, or blocking hormone action [1,2,3]. Given their involvement in the disruption of metabolic pathways and their potential contribution to the etiology of various disorders as well, EDs represent a major public health concern [1,4]. Surely a major challenge in the field of EDs is their structural diversity, which makes it difficult to predict whether a given compound will exhibit endocrine-disrupting activity [5]. In recent years, organic ultraviolet (UV) filters have gained increasing attention as emerging environmental EDs. Although their use has expanded in response to heightened awareness of the link between UV exposure and skin cancer, growing evidence indicates that several UV filters are ubiquitously present in the environment and may pose toxicological risks beyond their intended photoprotective function [6,7,8]. While UV-protective filters provide clear benefits for human health, their increasingly widespread use and the resulting ecological effects are raising growing concerns for the environment and public health [9]. Although an individual consumer applies only a small amount of sunscreen, the cumulative quantity used globally by all consumers counts several thousand tons. It is estimated that between 6000 and 14,000 tons of sunscreens enter the oceans each year, placing roughly 10% of the world’s coral reefs at risk of exposure [6,10]. For instance, some UV-filter compounds with maximum concentrations up to 40 μg/L have been found in river waters and swimming pools [11,12,13,14]. The widespread presence of these emerging contaminants in aquatic ecosystems also involves bioaccumulation and biomagnification phenomena, key processes in ecosystem pollution, raising concerns about their long-term ecological impact [7,11].

Among the most commonly used and effective organic UV filters there are butyl methoxydibenzoylmethane, commonly known as avobenzone (AVO) and 2-ethylhexyl salicylate (EHS), also known as octisalate or octyl salicylate [9,15]. Despite their regulatory approval and widespread use, toxicological and endocrine-related data for these compounds remain incomplete, particularly under conditions of chronic, low-dose exposure relevant to real-life environmental and human scenarios.

AVO is a dibenzoylmethane derivative used as a long-wavelength UVA filter. It is one of the most effective absorbers in the UVA region of the electromagnetic spectrum, providing protection against both the UVA1 and UVA2 ranges [16]. Currently, there is a finite number of UV filters approved by regulatory agencies, and AVO is one of those currently incorporated into sunscreen formulations. Indeed, being oil-soluble and of relatively low molecular weight (310.4 g/mol), AVO easily permeates the skin following topical application [9,16]. Dermal application of AVO resulted in very low systemic absorption and minimal urinary excretion, with only 0.012–0.016% of the applied dose detected in urine, under maximal use conditions. Plasma concentrations peaked shortly after application but declined to below detectable levels within 72 h [16]. Nevertheless, experimental evidence suggests that AVO and other organic UV filters can induce oxidative stress, endocrine-related effects, and early molecular alterations at concentrations below those causing overt cytotoxicity, indicating that classical toxicological endpoints may underestimate their biological impact [7,9,16].

EHS is one of the main components of the salicylate class, a light stabilizer and an effective UVB absorber insoluble in water, a characteristic that makes this filter suitable for use during bathing [9,15]. Indeed, the widespread use of sunscreens and other consumer products containing EHS can contribute to its potential bioaccumulation in the environment and its possible toxic effects on aquatic and terrestrial organisms. Moreover, EHS can be detected in rivers and wastewater effluents, since it exhibits low removal efficiency and high persistence during wastewater treatment compared to other UV filters [17]. The urinary excretion of EHS and its metabolites after dermal application is very low, ranging from 0.00001% to 0.0071% of the applied dose, much lower than after oral intake, where excretion reached about 0.1–0.3%. Cumulative excretion data suggest that only about 25–26% of the applied dose is eliminated within 24 h [15]. Overall, based on urinary excretion over 96 h, approximately 3% of the dermally applied EHS dose reached systemic circulation, consistent with in vitro studies showing low dermal bioavailability (~1.8–3.3%). However, its prolonged residence time in the skin suggests that repeated daily low-level exposure may lead to cumulative systemic exposure and potential long-term effects, raising concerns not fully addressed by current safety assessments [15,18]. The potential toxic effects of EDs on the central nervous system (CNS) have been demonstrated in both epidemiological and experimental studies [19]. Although the brain is protected by the blood–brain barrier (BBB), some EDs can cross it, reaching toxic concentrations and affecting neural development [20,21].

For AVO and EHS, however, data on early neurobiological effects, stress-responsive signaling, and epigenetic regulation remain limited.

Given the growing concern over the potential effects of these compounds, particularly in the CNS, this study aimed to assess the impact of AVO and EHS UV filters on cellular homeostasis and their possible implications for human health. To this end, this study examined the potential of AVO and EHS to affect key components of signaling cascades that regulate essential cellular functions such as proliferation, metabolism, differentiation, and survival. The modulation of these pathways was evaluated using the human neuroblastoma cell line SH-SY5Y to determine how these compounds could potentially impact adult brain health. This approach allowed for a detailed investigation of how AVO and EHS may modulate neuronal signaling pathways, providing insight into their potential neurotoxicity and impact on brain health.

By focusing on microRNA (miRNA) modulation and stress-responsive intracellular signaling pathways, this approach allows a mechanistic evaluation of early adaptive responses that may precede overt neurotoxicity or endocrine dysfunction.

## 2. Results

### 2.1. AVO and EHS UV Filters Do Not Affect Cell Proliferation and Reactive Oxygen Species Formation in Differentiated SH-SY5Y Cells

**Figure 1:**
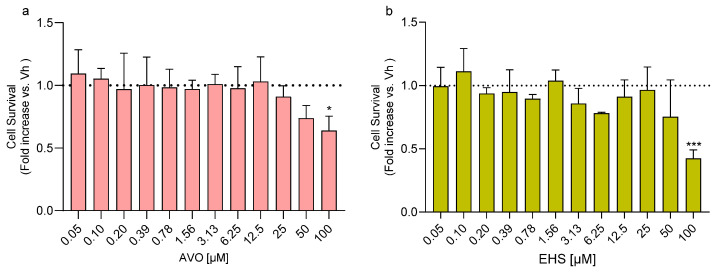

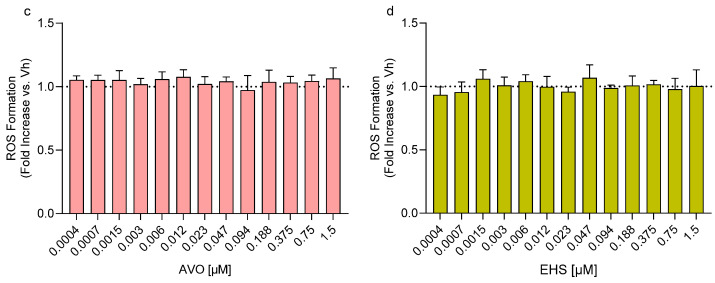
Effects of exposure to AVO and EHS on cell survival and ROS formation. Cells were differentiated with retinoic acid and treated for 48 h with UV filters. Cell viability (**a**,**b**) was evaluated by the reduction of resazurin to resorufin. ROS formation (**c**,**d**) was determined using the fluorescent probe H_2_DCF-DA. Data are expressed as fold increase versus the vehicle group and reported as mean ± SD of three independent experiments ((**a**): * *p* < 0.05 vs. 0.05; (**b**): *** *p* < 0.001 vs. 0.05; One-way ANOVA, post hoc Tukey test).

Differentiated SH-SY5Y cells were treated with increasing concentrations of AVO and EHS [0.05–100 µM] for 48 h in order to identify subcytotoxic doses. A significant reduction in cell viability was observed after exposure to 100 µM AVO or EHS for 48 h (Figure 1a,b). In parallel, the ability of these compounds to induce reactive oxygen species (ROS) formation was evaluated, and no significant changes were observed at any of the tested concentrations [0.0004–1.5 µM] (Figure 1c,d).

### 2.2. AVO and EHS UV Filters Induce miRNA Expression Modulation

**Figure 2:**
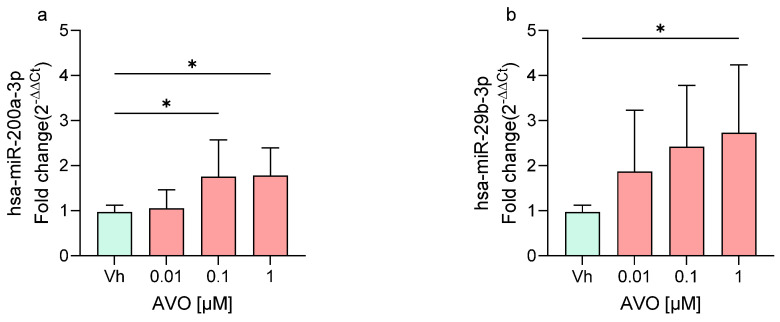

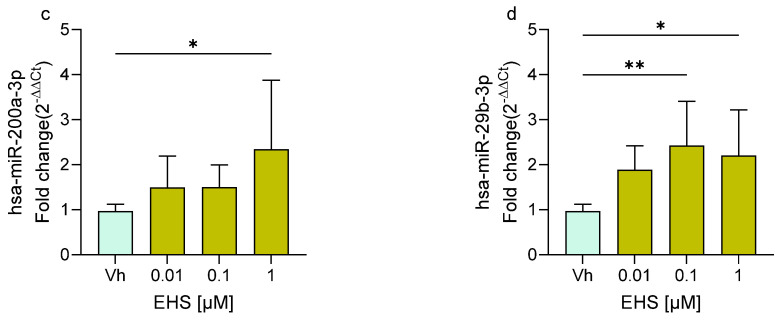
Differential expression of miR-200a-3p (**a**,**c**) and miR-29b-3p (**b**,**d**) in differentiated SH-SY5Y cells treated for 48 h with AVO or EHS [0.01, 0.1, 1 μM] by qRT-PCR. Quantitative analysis was performed by the 2^−ΔΔCt^ method and Vh samples were considered as the calibrator of the experiment. Data are expressed as fold increases and reported as mean ± SD of three independent experiments ((**a**–**c**): * *p* < 0.05 vs. Vh; (**d**): * *p* < 0.05 vs. Vh, ** *p* < 0.01 vs. Vh; One-way ANOVA, post hoc Dunnet test).

Following treatment of differentiated SH-SY5Y cells with AVO and EHS at concentrations of 0.01, 0.1, and 1 µM, the expression levels of two miRNAs, miR-200a-3p and miR-29b-3p, were quantified by qRT-PCR. These miRNAs, previously investigated following exposure to other EDs [22,23], are known to play key roles in neuronal maturation, neurodegeneration, and neuroinflammation [24,25,26]. As shown in Figure 2, exposure to both AVO (Figure 2a,b) and EHS (Figure 2c,d) significantly modulated the expression of these miRNAs compared to the vehicle group. Specifically, miR-200a-3p was significantly upregulated at 0.1 and 1 μM following AVO treatment (Figure 2a) and at 1 μM after EHS exposure (Figure 2c). Upregulation was most evident at the highest concentration tested (1 µM), with modest fold changes (~2-fold) across panels. MiR-29b-3p showed upregulation across concentrations for both AVO (Figure 2b) and EHS (Figure 2d), reaching significance following AVO (1 µM) and EHS (0.1 and 1 µM), but with no clear stepwise concentration response due to variability.

### 2.3. AVO and EHS UV Filters Alter Gene Expression Profiles

The expression of selected miRNA target genes was evaluated by qRT-PCR in differentiated SH-SY5Y cells treated with AVO and EHS [0.01, 0.1, and 1 µM] to assess UV filter–induced transcriptional changes relative to controls.

Specifically, the study investigated the modulation of *EGFR1*, *IGF1R-1*, and *PTEN* gene expression, all of which involved in the PI3K/Akt/mTOR signaling pathway that regulates cell growth, survival, metabolism, and protein synthesis. In addition, the expression of *BTG2*, a tumor suppressor gene, and *CDK6*, a cyclin-dependent kinase that controls the G1–S phase transition and promotes cell proliferation, was also evaluated [27,28].

**Figure 3:**
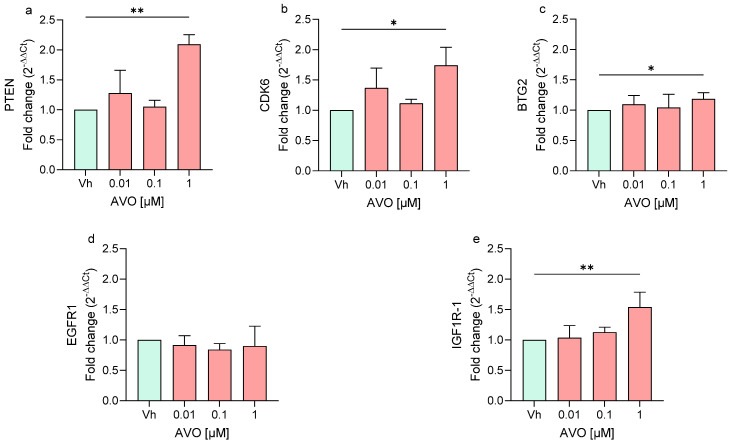
Differential expression of *PTEN* (**a**), *CDK6* (**b**), *BTG2* (**c**), *EGFR1* (**d**), *IGF1R* (**e**) genes in differentiated SH-SY5Y cells treated for 48 h with AVO [0.01, 0.1, 1 μM] by single-primer qRT-PCR. Quantitative analysis was performed by the 2^−ΔΔCt^ method and Vh samples were considered as the calibrator of the experiment. Data are expressed as fold increases and reported as mean ± SD of three independent experiments ((**a**,**e**): ** *p* < 0.01 vs. Vh; (**b**,**c**): * *p* < 0.05 vs. Vh; One-way ANOVA, post hoc Dunnet test).

In cells exposed to AVO, the treatment upregulated the expression of *PTEN*, *CDK6*, *BTG2*, and *IGF1R-1*, all significantly upregulated at the highest concentration (1 µM), while *EGFR1* expression showed no significant modulation (Figure 3).

**Figure 4:**
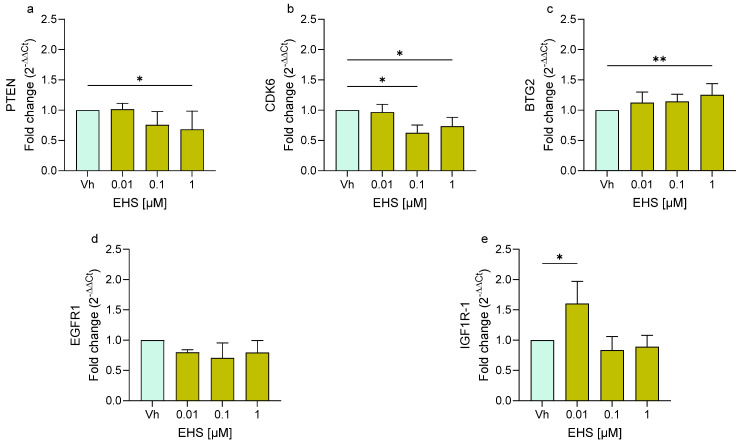
Differential expression of *PTEN* (**a**), *CDK6* (**b**), *BTG2* (**c**), *EGFR1* (**d**), *IGF1R* (**e**) genes in differentiated SH-SY5Y cells treated for 48 h with EHS [0.01, 0.1, 1 μM] by single-primer qRT-PCR. Quantitative analysis was performed by the 2^−ΔΔCt^ method and Vh samples were considered as the calibrator of the experiment. Data are expressed as fold increases and reported as mean ± SD of three independent experiments ((**a**,**b**,**e**): * *p* < 0.05 vs. Vh; (**c**): ** *p* < 0.01 vs. Vh; One-way ANOVA, post hoc Dunnet test).

Similarly, cells exposed to EHS (Figure 4) showed a significant upregulation of *BTG2* and *IGF1R-1* at the highest concentration used. In contrast, following 48 h of treatment, EHS induced the significant downregulation of *PTEN* and *CDK6*. On the other hand, EHS also did not show any variation in the expression of *EGFR1*.

In general, treatment with either AVO or EHS led to modest modulation of the expression of these genes, mainly at the highest concentration (1 µM), with effects that varied in magnitude, direction, and statistical significance between genes and compounds and no clear concentration–response patterns. Notably, the modest increases in miR-200a-3p and miR-29b-3p (Figure 2) did not consistently suppress their predicted targets, suggesting additional regulatory layers (e.g., other miRNAs or transcription factors).

### 2.4. AVO and EHS UV Filters Modulate the PI3K/Akt/mTOR and MAPK Signaling Pathways

**Figure 5:**
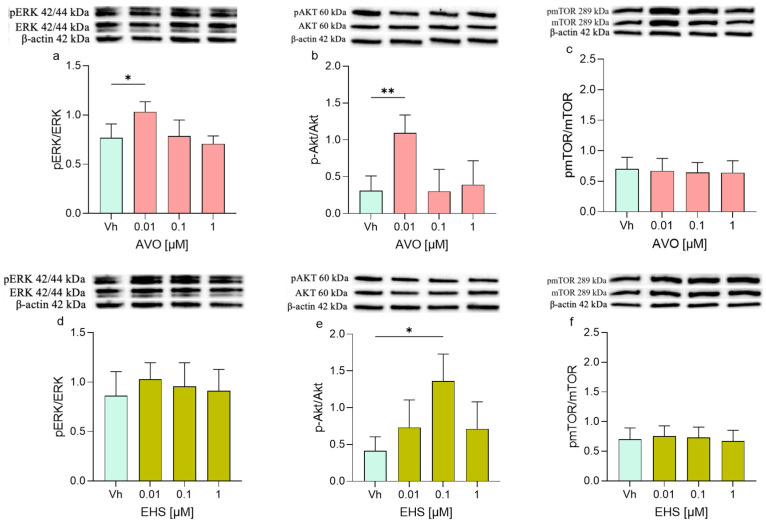
Differential phosphorylation of ERK, AKT, mTOR, in differentiated SHSY-5Y cells treated for 48 h with AVO (**a**–**c**) or EHS (**d**–**f**) [0.01, 0.1 and 1 µM]. The phosphorylation of ERK (**a**,**d**), AKT (**b**,**e**), and mTOR (**c**,**f**) were determined by Western blotting at 42/44, 60, and 289 kDa, respectively, and using total ERK, Akt, and mTOR as loading control. Top: cropped representative images of the protein of interest expressions. Bottom: quantitative analysis of the Western blotting results. The graphs show densitometry analysis of the bands appertaining to the protein of interest. Data are expressed as the ratio between the protein of interest and the corresponding loading control and reported as mean ± SD of three independent experiments ((**a**,**e**): * *p* < 0.05 vs. Vh; (**b**): ** *p* < 0.001 vs. Vh; One-way ANOVA, post hoc Dunnett test).

Differentiated SH-SY5Y cells were treated with AVO and EHS (0.01–1 µM), and protein expression was analyzed by Western blotting, focusing on key components of the PI3K/Akt/mTOR and MAPK/ERK signaling pathways involved in the regulation of cell proliferation, metabolism, survival, and apoptosis. Several proteins showed modulation in their expression levels after the treatment with AVO or EHS. Specifically, ERK phosphorylation increased after the treatment with both compounds, but its expression was significantly upregulated only following the exposure to AVO (Figure 5a). On the contrary, the treatment with both AVO and EHS was able to significantly increase the phosphorylation of pAKT (Figure 5b,e) but not pmTOR (Figure 5c,f).

### 2.5. AVO and EHS UV Filters Induced Modulation of EGFR/p53 and Bax/Bcl-2 Pathways

**Figure 6:**
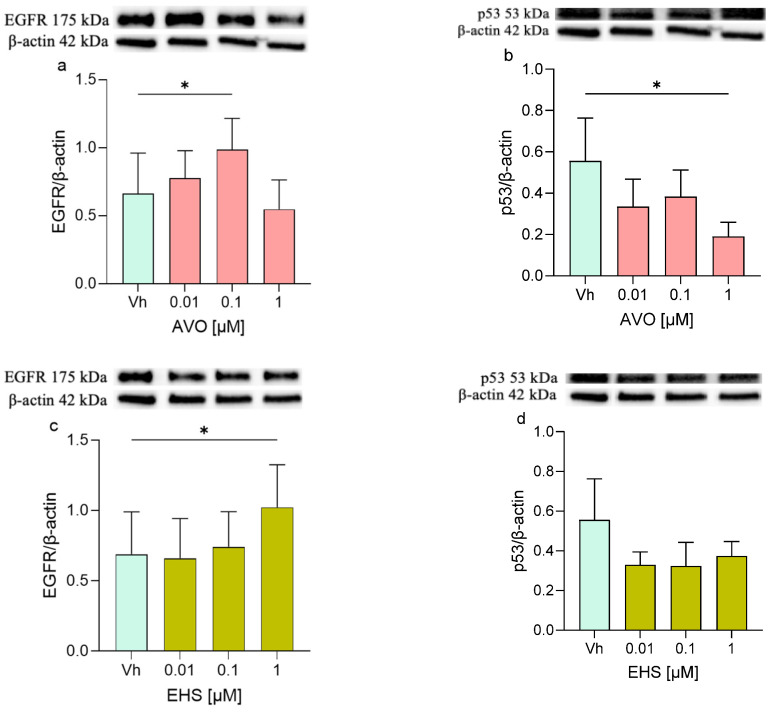
Differential expression of EGFR and p53 in differentiated SHSY-5Y cells treated for 48 h with AVO or EHS [0.01, 0.1 and 1 µM]. The expression of EGFR (**a**,**c**) and p53 (**b**,**d**) were determined by Western blotting at 175 and 53 kDa, respectively, and using β-actin (42 kDa) as loading control. Top: cropped representative images of the protein of interest expressions. Bottom: quantitative analysis of the Western blotting results. The graphs show densitometry analysis of the bands appertaining to the protein of interest. Data are expressed as the ratio between the protein of interest and the corresponding loading control and reported as mean ± SD of three independent experiments ((**a**–**c**): * *p* < 0.05 vs. Vh; One-way ANOVA, post hoc Dunnett test).

Furthermore, proteins involved in apoptotic and survival cellular pathways such as EGFR/p53 and the Bax/Bcl-2 were investigated. The EGFR protein showed increased levels in treated cells, with significant upregulation observed after treatment with AVO [0.1 µM] and with EHS [1 µM] (Figure 6a,c). Interestingly, the p53 expression mirrored this pattern, decreasing after both treatments, with statistical significance observed only at 48 h following AVO exposure. (Figure 6b,d).

**Figure 7:**
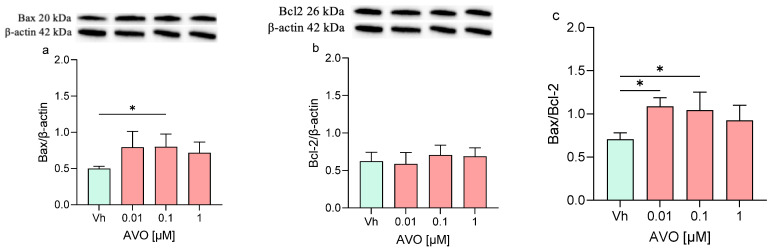

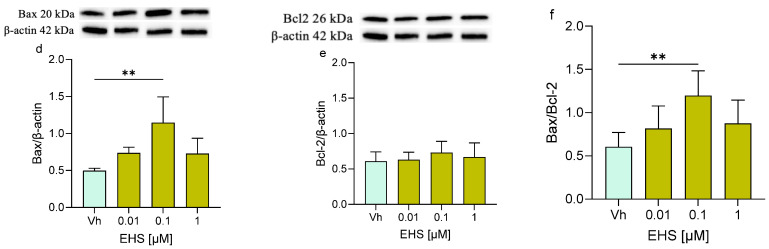
Differential expression of Bax and Bcl-2 in differentiated SH-SY5Y cells treated for 48 h with AVO or EHS [0.01, 0.1, 1 μM]. The proteins levels of Bax (**a**,**d**) and Bcl-2 (**b**,**e**) were determined by Western blotting at 20 and 26 kDa, respectively, and using β-actin (42 kDa) as loading control. The ratio between Bax and Bcl-2 (**c**,**e**). Top: cropped representative images of the protein of interest expressions. Bottom: quantitative analysis of the Western blotting results. The graphs show densitometry analysis of the bands appertaining to the protein of interest. Data are expressed as the ratio between the protein of interest and β-actin expression and reported as mean ± SD of three independent experiments ((**a**,**c**): * *p* < 0.05 vs. Vh; (**d**,**f**): ** *p* < 0.01 vs. Vh; One-way ANOVA, post hoc Dunnett test).

Finally, the expression levels of Bax and Bcl-2 and their ratio were assessed (Figure 7). Following the exposure to both AVO or EHS [0.1 µM], the expression of Bax was significantly increased but no significant modulation or modest modulation was detected in Bcl-2 levels, whose expression levels remained comparable to those of the control. Moreover, the Bax/Bcl-2 ratio was increased (with significant results at 0.01 and 0.1 µM), reflecting the trend of the single Bax and Bcl-2 proteins.

## 3. Discussion

Human exposure to EDs is widespread due to their extensive use in industrial, agricultural, and domestic applications, leading to environmental contamination, particularly of aquatic ecosystems, and lifelong accumulation, primarily through dietary intake [2,4,5,29]. A key long-term effect of EDs exposure may involve the induction of epigenetic alterations, including modulation of miRNAs expression [30]. MiRNAs are abundantly expressed in the CNS, where they play crucial roles in neuronal function; indeed, dysregulated miRNAs have been implicated in several human diseases [26,31].

UV filters, widely used in sunscreens and cosmetics, represent an emerging class of potential EDs with weak estrogenic activity demonstrated mainly in vitro for some compounds, linked to reproductive effects in animal models but with limited evidence for neurological disorders [5,6,7,19]. For AVO and EHS specifically, in vitro data indicate low estrogenic receptor (ER) binding affinity and weak endocrine potential, without established links to clinical endocrine disruption or neurotoxicity in vivo [32,33]. Some UV filters can cross the BBB and affect brain endpoints, raising concern that lipophilic filters like AVO and EHS may pose risk to CNS hormone signaling, which our study addresses mechanistically in neuronal cells [21].

This study investigated epigenetic regulation in differentiated SHSY-5Y neuronal cells exposed for 48 h to three subcytotoxic concentrations (0.01, 0.1, and 1 µM) of AVO or EHS, focusing on miRNA expression. While subtoxic concentrations of both AVO and EHS do not affect cell proliferation, differentiated SH-SY5Y cells respond to the exposure by activating cell survival pathways. The in vitro concentrations used (0.01, 0.1, and 1 µM) for both AVO and EHS are higher than human systemic plasma levels after topical sunscreen application, which generally lie in the low nanomolar range (≈1–5 nM) for both compounds, even under maximal-use conditions [34,35,36]. The use of micromolar concentrations in vitro is well-established for organic UV filters and is justified for investigating cellular effects and worst-case exposure scenarios that cannot be directly extrapolated from plasma pharmacokinetic data alone [36,37]. Specifically, two miRNAs, miR-200a-3p and miR-29b-3p, were examined because of their established involvement in neuronal maturation, neurodegeneration, and neuroinflammation [24].

MiR-200a-3p, a member of the miR-200 family, regulates cell differentiation, apoptosis, proliferation, signaling pathways such as PI3K/AKT and MAPK, and has been implicated in several human diseases, including cancer and AD. Its role in neurodegeneration remains controversial, likely reflecting its multiple downstream targets and the complexity of the networks it controls [24].

MiR-29b-3p is widely expressed, with particularly high levels in the brain, where it contributes to neuronal differentiation and survival. It is a key regulator of intrinsic apoptosis, modulating the balance between pro-apoptotic proteins, such as Bax, and anti-apoptotic proteins, such as Bcl-2, and altered miR-29b expression has been reported in AD and other CNS disorders, suggesting that maintaining appropriate miR-29b-3p levels is critical for cellular homeostasis and neuroprotection [25,26]. Experimental data showed modest upregulation of miR-200a-3p and miR-29b-3p following treatment with either AVO or EHS. Because both miRNAs participate in the regulation of apoptosis and cell survival, their increased expression indicates that cells recognize the treatments as stressors and respond by enhancing miRNAs associated with apoptotic pathways and stress-related pathways, potentially shifting cell balance toward apoptosis [24,25].

This response was partly mirrored at the gene-expression level. After miRNAs modulation, several target genes, including *IGF1R-1*, *EGFR1*, *PTEN*, *BTG2*, and *CDK6*, were analyzed to further characterize the cellular response. Contrary to the expectation of miRNA-mediated repression, AVO induced general upregulation of *PTEN*, *CDK6*, *BTG2*, and *IGF1R-1* (mainly at 1 µM), while EHS showed mixed effects (downregulation of *PTEN/CDK6*, upregulation of *BTG2/IGF1R*). This divergence underscores the complexity of miRNA regulation in stress contexts. EGFR1 and IGF1R-1 are tyrosine kinase receptors that are key components of the PI3K/AKT/mTOR axis, which regulates cell growth, proliferation, differentiation, and survival [27,38,39]. The activation of this pathway by growth factors, such as IGF-1 or EGF, promotes PI3K-dependent generation of PIP3, recruitment and activation of AKT, and downstream stimulation of mTOR and other effectors, whereas PTEN, a suppressor, counteracts this signaling by converting PIP3 back to PIP2 [27,40].

BTG2, an antiproliferative tumor suppressor, and CDK6, a cyclin-dependent kinase controlling G1-S phase progression, are not core components of the PI3K/AKT, but can be indirectly modulated by this pathway. *BTG2* is a p53-responsive gene whose expression decreases when PI3K/AKT activity inhibits p53, while PI3K/AKT activation favors cell-cycle progression and is typically associated with increased CDK6 expression [28,41,42].

On the basis of miRNA upregulation, a reduction in the expression of target genes was expected. This pattern was more evident after EHS exposure, whereas AVO elicited a more complex profile. AVO treatment induced a general increase in *IGF1R-1*, *PTEN*, *CDK6*, and *BTG2* expression. The concurrent upregulation of pro-proliferative genes (*CDK6*, *IGF1R-1*) and anti-proliferative genes (*PTEN*, *BTG2*) may reflect a finely tuned adaptive response, in which cells activate survival and growth pathways while simultaneously constraining uncontrolled proliferation [27,28,41]. In contrast, EHS treatment led to significant upregulation of *IGF1R* and *BTG2* and significant downregulation of *PTEN* and *CDK6*. Given the functions of CDK6 and BTG2, this pattern is consistent with an antiproliferative response to a harmful stimulus, with increased *BTG2* and reduced *CDK6* indicating cell-cycle arrest and diminished proliferation [28,41].

Although increased PTEN levels would typically be expected to inhibit PI3K/Akt signaling, given its role as a negative regulator of the pathway, the transient decrease in PTEN observed with EHS may not necessarily indicate activation of proliferative signaling, but rather a protective adaptation. By reducing *PTEN* and increasing *IGFR-1*, cells may preserve a degree of PI3K/AKT activity as a short-term survival mechanism, while simultaneously repressing growth-promoting genes (such as *CDK6*) and enhancing antiproliferative genes (such as *BTG2*). In this way, cells appear to promote survival but limit proliferation, adjusting signaling networks to restore homeostasis under stress [27,28,41,42].

Under both treatments, the upregulation of miR-200a-3p and miR-29b-3p would generally be expected to suppress their targets. Whereas EHS largely conforms to a miRNA-dependent repression model, AVO seems to induce a more multilayered regulatory response in which target genes are not uniformly downregulated. This discrepancy suggests the involvement of additional mechanisms, including other miRNAs, transcription factors, or post-transcriptional regulation, that may counterbalance the inhibitory actions of miR-200a-3p and miR-29b-3p. Given that miRNAs can simultaneously modulate numerous genes across interconnected pathways, their net effects can be context-dependent and occasionally divergent [23,43].

In this framework, miRNA-driven gene modulation points to a dual cellular strategy that promotes both pro-survival and antiproliferative responses. Through this dual role, miRNAs can support short-term survival while still enabling growth arrest or apoptosis when stress becomes irreparable [23,43].

Protein analyses were performed to validate changes at the functional level, focusing on key proteins within the PI3K/AKT/mTOR, p53, and MAPK/ERK pathways. After both AVO and EHS exposure, increased ERK phosphorylation and AKT levels were detected, with no significant change in pmTOR. A modest increase in EGFR expression was observed, together with reduced p53 levels. Bax expression was elevated, Bcl-2 remained largely unchanged, and the Bax/Bcl-2 ratio increased.

These data indicate that cells activate PI3K/AKT and MAPK/ERK pro-survival signaling in response to AVO and EHS, as reflected by AKT and ERK activation and a mild rise in EGFR. The absence of substantial pmTOR modulation suggests that the effects of these UV filters are more pronounced upstream of mTOR or are buffered by compensatory controls that maintain mTOR activity near baseline [27,44].

The enhanced phosphorylation of AKT and ERK coincided with reduced p53, supporting an early adaptive, pro-survival program aimed at counteracting stress and maintaining viability. This response likely reflects dynamic crosstalk between pro-survival signaling and p53-mediated cell death, with AKT playing a central role by activating MDM2, promoting p53 degradation, and thereby limiting p53-dependent cell-cycle arrest and apoptosis [27,45].

This survival-oriented state is generally transient in nature. In early phases, AKT-mediated suppression of p53 favors repair and recovery, but under prolonged or more intense stress, this balance can tip, leading to reactivation of p53 and a switch toward growth arrest, autophagy, or apoptosis. This biphasic pattern, characterized by initial pro-survival signaling followed by delayed p53 activation, is a recognized hallmark of cellular stress responses [45].

The increased Bax expression and elevated Bax/Bcl-2 reflects early or partial activation of stress-related apoptotic signaling. Although Bax upregulation is often linked to p53 activation, Bax can also be induced through p53-independent mechanisms. Several stress-responsive pathways can modulate Bax independently of p53, and early Bax induction may precede full p53 activation, arising from parallel signaling cascades [46].

Altogether, these findings support a model in which UV-filter exposure initially elicits a compensatory survival program, characterized by AKT and ERK activation and p53 suppression, allowing cells to transiently cope with stress. As damage accumulates or persists, this equilibrium may fail, leading to engagement of apoptotic pathways. In the present conditions, the data suggest a potential shift toward p53-independent mitochondrial apoptosis, as indicated by Bax upregulation and the increased Bax/Bcl-2 ratio [45,46].

This intricate interplay between p53, AKT/mTOR, ERK, and additional stress-responsive pathways illustrates the complexity of the cellular response to AVO and EHS and underscores the pivotal role of the p53–AKT/mTOR axis in determining cell fate. Cells continuously recalibrate survival and death decisions in response to environmental challenges, and the integrated miRNA, gene, and protein data presented here indicate that AVO and EHS are perceived as stressors that trigger early PI3K/AKT and MAPK/ERK activation, followed by a potential shift toward p53-independent mitochondrial apoptosis once stress exceeds the capacity of adaptive defenses [45].

Thus, our integrated miRNA, gene, and protein data suggest that AVO and EHS are perceived as stressors that engage both pro-survival and pro-apoptotic pathways, but further work is needed to determine whether and when this balance actually progresses to measurable apoptosis in vitro or in vivo.

## 4. Materials and Methods

### 4.1. Chemicals

AVO (CAS Number: 70356-09-1) and EHS (CAS Number: 118-60-5) were supplied by Fluorochem Ltd. (Glossop, Derbyshire, UK). H_2_DCF-DA, eosin, retinoic acid, leupeptin, phenylmethylsulfonyl fluoride (PMSF), β-mercaptoethanol, sodium dodecyl sulfate (SDS), dimethyl sulfoxide (DMSO), Tris·HCl and β-actin antibody were obtained from Merck Life Science S.r.L. (Darmstadt, Germany). Dulbecco’s Modified Eagle Medium (DMEM), penicillin and streptomycin mixture, glutamine, trypsin–EDTA, fetal bovine serum (FBS), Dulbecco phosphate-buffered saline (DPBS), Hanks’ balanced salt solution (HBSS), and antibodies by Cell Signaling Inc. (p-ERK, total ERK, p-AKT, total AKT, phospho-mTOR (p-mTOR), mTOR, EGFR, p53, Bax, and Bcl-2) were purchased from Euroclone S.p.A (Pero, MI, Italy). AlamarBlue HSTM, Pure link RNA mini kit, High-Capacity RNA-to-cDNA kit, PowerUp SYBR Green Master Mix assay were purchased from Thermo Fisher Scientific (Waltham, MA, USA). The Mir-X miRNA First-Strand Synthesis and TB green qRT-PCR assay were obtained from Takara (Mountain View, CA, USA). Quick Start™ Bradford 1× Dye Reagent, 4× Laemmli buffer, Clarity Western ECL Substrate was sourced from Bio-Rad Laboratories S.r.l. (Hercules, CA, USA). Horseradish peroxidase–conjugated secondary antibodies (goat anti-mouse and goat anti-rabbit) were purchased from Jackson ImmunoResearch Europe Ltd. (Cambridgeshire, UK). All of the reagents used in this study were of analytical grade and the highest purity commercially available.

### 4.2. Cell Culture

#### 4.2.1. SH-SY5Y

The human neuronal SH-SY5Y cells were obtained from the Lombardy and Emilia Romagna Experimental Zootechnic Institute (Brescia, BS, Italy). Cells were routinely maintained at 37 °C in a humidified atmosphere with 5% CO_2_ and cultured in phenol red-free DMEM supplemented with 10% FBS, 2 mM glutamine, 50 U/mL penicillin, and 50 μg/mL streptomycin.

#### 4.2.2. SH-SY5Y Differentiation

SH-SY5Y cells were plated in six-well culture plates at a density of 1 × 10^5^ cells per well in phenol red-free DMEM supplemented with 10% FBS. Five hours after plating, the culture medium was replaced, and cells were exposed to retinoic acid at a final concentration of 10 µM in phenol red-free DMEM containing 2% FBS. The differentiation procedure was performed twice, with treatments repeated every 48 h.

### 4.3. AVO and EHS Treatments

Upon completion of the differentiation protocol, SH-SY5Y cells were exposed for 48 h to increasing concentrations of the test compounds (AVO or EHS). Treatments were carried out in phenol red-free DMEM supplemented with 2% FBS. Stock solutions (100 mM) were prepared in DMSO and subsequently diluted in culture medium to achieve the desired concentrations, ensuring a final DMSO content not exceeding 0.1%.

### 4.4. Determination of Neuronal Viability

Neuronal viability was evaluated using the AlamarBlue™ assay, which is based on the reduction of resazurin to resorufin [47]. Differentiated SH-SY5Y cells were seeded into 96-well plates at a density of 2 × 10^3^ cells per well and allowed to adhere for 24 h. Cells were then exposed to increasing concentrations of AVO or EHS (0.05–100 µM) for 48 h at 37 °C in 5% CO_2_. Following treatment, 10 µL of the 10× AlamarBlue™ reagent was added to each well and incubated for 1 h under the same conditions. Fluorescence was measured using a multilabel plate reader (GENios, TECAN^®^, Mannedorf, Switzerland) at an excitation wavelength of 570 nm and reference filter at 690 nm. Results are reported as fold changes in cell viability (mean ± SD) relative to vehicle-treated control cells (DMSO < 0.1%).

### 4.5. Determination of ROS Formation

Intracellular ROS levels were measured to assess the neuronal redox status, as previously reported [22]. Differentiated SH-SY5Y cells were exposed to UV filters (0.0004–1.5 µM) for 48 h at 37 °C in 5% CO_2_. Following treatment, the medium was removed and cells were incubated with 100 µL of H_2_DCF-DA (10 μg/mL). After 30 min, the probe was discarded and intracellular ROS production was quantified) using a multilabel plate reader (GENios, TECAN^®^, Mannedorf, Switzerland) at an excitation wavelength of 485 nm and an emission wavelength of 535 nm. Results are expressed as the fold changes in ROS levels (mean ± SD) relative to vehicle-treated control cells (DMSO < 0.1%).

### 4.6. Pellet Preparation

After 48 h of exposure to AVO or EHS [0.01, 0.1, 1 µM], cells were harvested using a 0.02% EDTA-trypsin solution. The resulting cell suspension was collected into 15 mL tubes and centrifuged at 1400 rpm for 8 min. The supernatant was discarded, and the cell pellet was washed with 1 mL of D’PBS, transferred to a 1.5 mL microcentrifuge tubes, and centrifuged at 6000 rpm for 5 min at 4 °C. Following removal of the supernatant, cell pellets were stored at −80 °C until further analysis.

### 4.7. RNA Extraction

A fraction of the collected cell pellets was processed for total RNA isolation using the PureLink RNA Mini Kit (Thermo Fisher Scientific, Waltham, MA, USA), following the manufacturer’s instructions and as previously reported [23]. Briefly, cell pellets were lysed on ice in lysis buffer supplemented with 1% β-mercaptoethanol, mixed with 70% ethanol, and loaded onto the spin cartridge provided with the kit. After successive washing steps, total RNA was eluted with RNase-free water. RNA concentration and purity were determined by spectrophotometric analysis using a NanoDrop^TM^ instrument (Thermo Fisher Scientific), and samples were stored at −80 °C until further use.

### 4.8. Analysis of miRNAs Expression and Quantitative Real-Time PCR (qRT-PCR)

Total RNA was reverse transcribed into cDNA for miRNA analysis using the Mir-X miRNA First-Strand Synthesis and TB Green qRT-PCR assay (Takara), according to the manufacturer’s instructions. The resulting cDNA was stored at −20 °C until use. MiRNA expression levels were quantified using the same system, with miR-U6 serving as the endogenous control. Analyses were performed on differentiated SH-SY5Y cells treated with AVO or EHS at concentrations of 0.01, 0.1, 1 µM, 100 nM, or 1 µM for 48 h at 37 °C in 5% CO_2_. Quantitative PCR was carried out on a QuantStudio™ 7 Flex Real-Time PCR System (Thermo Fisher Scientific), and each sample was analyzed in triplicate. Relative miRNA expression was calculated using the 2^−ΔΔCt^ method, with vehicle-treated samples used as the calibrator. Primer sequences are reported in Table S1. MiRNA-specific primers used for qPCR were designed based on sequences obtained from the miRBase genome browser [48].

### 4.9. Gene Expression Analysis by RNA Reverse Transcription and qRT-PCR

For each sample, total RNA was reverse-transcribed into cDNA for gene expression analysis using the High-Capacity RNA-to-cDNA kit (Thermo Fisher Scientific), following the manufacturer’s instructions. The resulting cDNA was stored at −20 °C until analysis. Relative gene expression was subsequently assessed by quantitative real-time PCR (qRT-PCR) using the PowerUp SYBR Green Master Mix assay (Thermo Fisher Scientific). The expression levels of *PTEN*, *CDK6*, *BTG2*, *EGFR1*, *IGF1R-1* were quantified, with *GAPDH* used as the endogenous reference gene (Table S2). Primer sequences used for qPCR were designed based on the Ensembl genome browser and the Benchling platform [48]. Analyses were performed on differentiated SH-SY5Y cells previously treated with AVO or EHS at concentrations of 0.01, 0.1, or 1 µM for 48 h at 37 °C in 5% CO_2_. Each reaction was carried out in triplicate, and elative gene expression was calculated using the 2^−ΔΔCt^ method. Vehicle-treated samples were used as the calibrator.

### 4.10. Western Blotting

The phosphorylation status of ERK, AKT and mTOR, as well as the expression of EGFR, p53, Bax, and Bcl-2, was analyzed by Western blotting, as previously described [22]. Briefly, pellets of retinoic acid-differentiated SH-SY5Y cells treated with AVO or EHS were resuspended in complete lysis buffer supplemented with leupeptin (2 µg/mL), PMSF (100 µg/mL) and a protease/phosphatase inhibitor cocktail (100×). Following protein extraction, the total protein concentration was determined using the Bradford method, and lysates were stored at −80 °C until analysis. For sample preparation, 4× Laemmli sample buffer was supplemented with β-mercaptoethanol and mixed with protein extracts at a 1:3 ratio to obtain a final 1× concentration. Samples were denatured at 95 °C for 5 min and stored at −20 °C until electrophoresis. Equal amounts of protein (30 µg) were separated on 4–15% SDS–polyacrylamide gels (Bio-Rad Laboratories S.r.l.) and transferred onto 0.45 µm nitrocellulose membranes. Membranes were incubated with primary antibodies against p-ERK, p-AKT, p-mTOR, EGFR, p53, Bax, and Bcl-2 (all diluted 1:1000), followed by incubation with appropriate secondary antibodies. Immunoreactive bands were visualized using enhanced chemiluminescence (ECL) reagents. Membranes were subsequently stripped and reprobed with antibodies against total ERK, AKT, mTOR, and β-actin (all 1:1000; raw images are shown in the Supplementary Materials). Band intensities were quantified by densitometric analysis using Quantity One software 4.2.3 (Bio-Rad Laboratories S.r.L.). Data are presented as the ratio of phosphorylated to total protein levels or as the ratio of the target protein to β-actin expression.

### 4.11. Statistical Analysis

The data obtained were analyzed using GraphPad Prism 10 (GraphPad Software, La Jolla, CA, USA). Differences between experimental groups were assessed using one-way ANOVA followed by Tukey’s (cell survival and ROS formation) or Dunnett’s post hoc test. The results were evaluated based on the *p*-value, which indicates whether the observed increase or decrease relative to the control sample is statistically significant. Results were finally presented as the mean ± SD for each group compared to the Vh group, and a *p*-value less than 0.05 was considered indicative of statistical significance.

## 5. Conclusions

In conclusion, EDs, including several UV filters, represent a significant environmental and public health concern due to their widespread occurrence, persistence, and ability to interfere with critical hormonal and physiological processes. Growing evidence links ED exposure to alterations in growth, metabolism, reproduction, and neurodevelopment, underscoring the need to reduce environmental contamination and human exposure, particularly during vulnerable developmental windows [1,4].

Although most UV filters currently used in sunscreens are regarded as safe at permitted concentrations, their continuous release into the environment raises concerns about bioaccumulation and potential endocrine activity.

Under the experimental conditions employed, AVO and EHS did not induce cytotoxicity in differentiated SH-SY5Y cells but elicited modest and heterogeneous modulation of miRNA expression and stress-related signaling pathways. The lack of a consistent concentration–response relationship and the limited concordance between miRNA modulation and target gene expression indicate that these responses reflect early, adaptive cellular signaling rather than commitment to apoptosis or overt neurotoxicity.

Although the activation of PI3K/AKT and MAPK/ERK pathways, together with changes in Bax/Bcl-2 signaling, suggests an engagement of stress-responsive mechanisms, these molecular alterations were not accompanied by functional evidence of programmed cell death. Moreover, the compound-specific nature of the responses underscores the difficulty of extrapolating class-level conclusions to individual UV filters.

Taken together, the present findings do not support direct extrapolation of subcytotoxic exposure to AVO or EHS toward neurotoxicity or endocrine disruption. Instead, they provide a hypothesis-generating framework for future investigations aimed at assessing long-term exposure scenarios, additional regulatory layers, and functional neuronal outcomes.

## Data Availability

The original contributions presented in this study are included in the article/Supplementary Material. Further inquiries can be directed to the corresponding authors.

## Supplementary Materials

The following supporting information can be downloaded at https://www.mdpi.com/article/10.3390/ijms27031134/s1.
