# Supplementary material for: Subcytotoxic Exposure to Avobenzone and Ethylhexyl Salicylate Induces microRNA Modulation and Stress-Responsive PI3K/AKT and MAPK Signaling in Differentiated SH-SY5Y Cells
Source: Int J Mol Sci. 2026 Jan 23;27(3):1134. doi: 10.3390/ijms27031134 (PMC12897653; doi:10.3390/ijms27031134)
Supplement: Supplementary file 1 [file ijms-27-01134-s001.zip › ijms-4087079-supplementary.pdf]

Table S1. Primers sequence for miRNA validation used for qPCR

miRNA	Primer Sequence (5′-3′)	Utilization
miR-29b-3p	Forward: TAGCACCATTTGAAATCAGTGTT	qPCR
	Reverse: mRQ 3' Primer (TaKaRa)	
miR-200a-3p	Forward: TAACACTGTCTGGTAACGATGT	qPCR
	Reverse: mRQ 3' Primer (TaKaRa)	
Mir-U6	Forward: mRQ 5' Primer (TaKaRa)	qPCR
	Reverse: mRQ 3' Primer (TaKaRa)	

Table S2. Primers sequence for gene expression used for qPCR

Gene	Primer Sequence (5′-3′)	Utilization
PTEN	Forward: GCGGAACTTGCAATCCTCAG	qPCR
	Reverse: AACTTGTCTTCCCGTCGTGT	
BTG2	Forward: GGCACTCACAGAGCACTACA	qPCR
	Reverse: GGGGTCCATCTTGTGGTTGA	
EGFR1	Forward: AGTGTGATCCAAGCTGTCCC	qPCR
	Reverse: ACTGCTGGGCACAGATGATT	
CDK6	Forward: TGGATCTCTGGAGTGTTGGC	qPCR
	Reverse: GGGAGTCCAATCACGTCCAA	
IGF1R-1	Forward: ATGCTCCAAGGATGCACCAT	qPCR
	Reverse: CTCGATGAGCCCCATGAAGT	
GAPDH	Forward: TCAAGAAGGTGGTGAAGCAG	qPCR
	Reverse: GTTGAAGTCAGAGGAG	
